# Systemic immune inflammation index guides machine learning for rapid TTP diagnosis: a retrospective cohort study

**DOI:** 10.3389/fmed.2025.1599999

**Published:** 2025-10-16

**Authors:** Zhenqi Liu, Xu Ye

**Affiliations:** Department of Hematology, The Second Affiliated Hospital of Guangzhou Medical University, Guangzhou, China

**Keywords:** thrombotic thrombocytopenic purpura, systemic immune-inflammation index, inflammatory biomarkers, machine learning, early diagnosis, logistic regression

## Abstract

Thrombotic thrombocytopenic purpura (TTP) is a rare, life threatening thrombotic microangiopathy that requires prompt diagnosis to reduce mortality. However, its early identification is often hindered by delayed ADAMTS13 testing, particularly in low resource settings. In this study, we developed a machine learning–based model using readily available inflammatory markers, including systemic immune inflammation index (SII), platelet to lymphocyte ratio (PLR), and platelet neutrophil product (PPN), to distinguish TTP from immune thrombocytopenia (ITP). A retrospective analysis of 196 hospitalized patients was conducted, and eight machine learning models were trained and compared. Logistic regression achieved the best performance (AUC = 0.78), with SII identified as the most influential predictor. While the PLASMIC score remains a widely used tool with higher diagnostic accuracy (AUC = 0.92), our model relies only on routine blood tests and offers a fast, accessible alternative for early risk stratification. These findings suggest that composite inflammatory markers combined with machine learning can assist in the rapid triage of suspected TTP cases, especially in emergency or resource-limited environments.

## 1 Introduction

Timely diagnosis of thrombotic thrombocytopenic purpura (TTP) remains challenging due to its rarity, clinical heterogeneity, and reliance on delayed ADAMTS13 assays. Research indicates that the principal mechanism involves a shortage of von Willebrand factor cleaving protease, identified as a disintegrin and metalloproteinase with thrombospondin type 1 motif 13 (ADAMTS13), resulting in the formation of platelet rich thrombi in microvessels (1–3). Owing to its varied and intricate clinical characteristics, the main diagnosis depends on the detection of significantly decreased ADAMTS13 activity (4). However, the accessibility of ADAMTS13 detection is influenced by various factors, such as the local medical environment, economic resources, clinician awareness, and the detection cycle, which might result in missed opportunities for timely diagnosis and treatment of TTP. Recent studies have identified that persistent inflammatory responses may result in aberrant aggregation of von willebrand factor (VWF) and activity defects or ADAMTS13 dysfunction, thus leading to platelet consumption and diminished platelet amount (5). Moreover, inflammation is not only a causative component of microangiopathic hemolytic anaemia but can also facilitate disease progression by producing micro-thrombosis and damaging red blood cells (6). Consequently, inflammatory factors in the blood may be closely related to TTP. However, few studies have investigated the role of routine inflammatory markers in predicting the occurrence of TTP, particularly in the context of rapid differential diagnosis.

Composite inflammatory markers help mitigate random fluctuations from individual variability, enhance disease specificity and predictive power, and provide a more comprehensive assessment of the systemic inflammatory state thereby improving diagnostic stability and accuracy in clinical practice (7, 8). Among these, the systemic immune-inflammation index (SII), calculated as the product of the platelet count and the neutrophil to lymphocyte ratio, reflects the dynamic interplay between inflammation and immune response. SII has been widely studied as a prognostic biomarker in malignancies, neuropsychiatric disorders, dermatological diseases and serum iron (9–14).

Notably, SII integrates three key cellular components (platelets, neutrophils, and lymphocytes) that are critically involved in the pathogenesis of thrombotic thrombocytopenic purpura (TTP), particularly through their roles in thrombus formation and immune dysregulation. Despite its growing recognition in other systemic diseases, the diagnostic value of SII in immune mediated hematologic disorders such as TTP has received little attention. Investigating SII in this context may thus uncover a simple, accessible, and potentially powerful biomarker for early TTP identification. Similarly, platelet and neutrophil production (PPN) has been explored in cancer, endocrine disorders, and autoimmune diseases, reflecting the relationship between inflammation and thrombosis (15). The platelet to lymphocyte ratio (PLR) serves as another surrogate marker of platelet activation and prothrombotic status (16, 17). While these composite indices have demonstrated clinical utility in various disease contexts, their specific roles in hematological disorders (particularly in differentiating TTP from conditions with overlapping presentations like immune thrombocytopenia (ITP)) remain underexplored and warrant systematic evaluation.

Current reliance on ADAMTS13 activity assays presents significant limitations due to prolonged turnaround time (typically 24–72 h) and limited accessibility in many clinical settings (1). To address this diagnostic gap, we propose the first machine learning based model integrating SII, PPN, and PLR (readily obtainable from routine blood tests) for rapid and practical TTP risk stratification.

## 2 Methods

### 2.1 Study population

**Figure 1:**
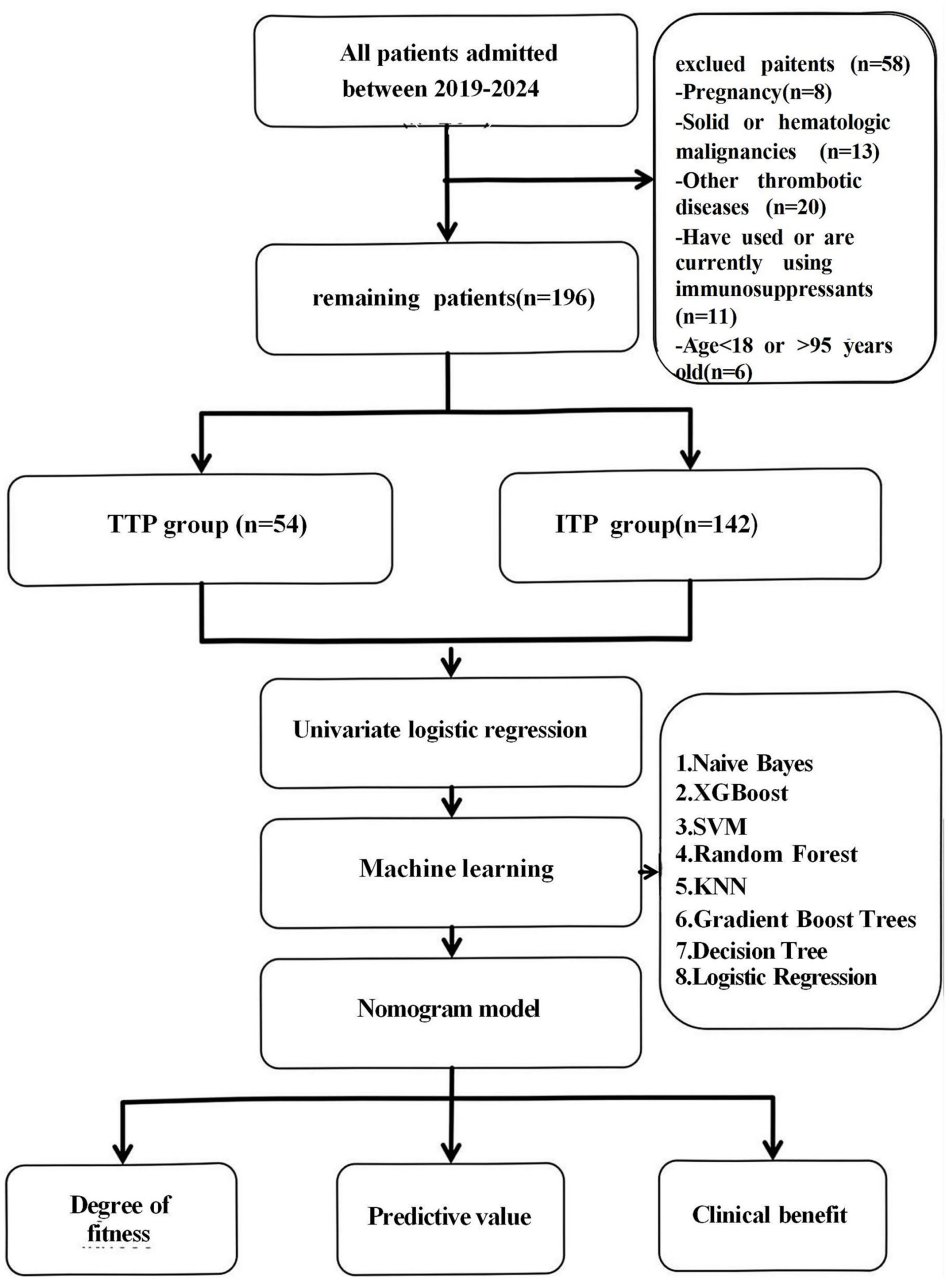
Flowchart. A total of 196 patients were retrospectively enrolled and categorized into the TTP (*n* = 54) and ITP (*n* = 142) groups. Candidate risk factors were analyzed using univariate logistic regression, and eight machine learning models were developed using selected variables. The best-performing model was selected based on AUC, clinical interpretability, calibration, and net benefit.

Both TTP and Immune Thrombocytopenia (ITP) present with thrombocytopenia in clinical practice, which may be related to inflammatory factors. In order to improve the specificity of TTP diagnosis, highlight the characteristics of TTP related inflammatory factors, and improve the clinical practicality of the prediction model, we selected TTP and ITP patients as control subjects for the study. This retrospective investigation was performed in the Second Affiliated Hospital of Guangzhou Medical University in Guangdong Province, China. A total of 254 consecutive patients with thrombocytopenia admitted between May 22, 2019, and May 22, 2024, were included. This study adhered to the principles of the Declaration of Helsinki and received approval from the Clinical Research and Application Ethics Committee of the Second Affiliated Hospital of Guangzhou Medical University. The inclusion criteria consisted of participants aged ≥18 years and<95 years, who underwent regular blood tests within 24 h of admission; and patients objectively diagnosed with TTP or ITP upon initial admission, possessing comprehensive clinical data. The exclusion criteria encompassed pregnant women, individuals with solid or haematologic malignancies, those with other thrombotic diseases, and patients using immunosuppressants (18). According to the “Chinese Guidelines for the Diagnosis and Treatment of Primary Immune Thrombocytopenia in Adults (2020 Edition).” The diagnosis of ITP is mainly based on clinical exclusion, requiring a decrease in platelet count, generally no splenomegaly, and bone marrow cell morphology characterized by increased or normal megakaryocytes with maturation disorders etc. (19). According to the “Chinese Guidelines for the Diagnosis and Treatment of Thrombotic Thrombocytopenic Purpura (2022 Edition).” The diagnosis of TTP, after excluding other thrombotic microangiopathy, includes: significantly decreased platelet count, fragmented red blood cells and increased reticulocyte ratio in peripheral blood smear; increased blood bilirubin, significantly increased lactate dehydrogenase (LDH), increased blood urea nitrogen and creatinine, and detection of ADAMTS13 activity and inhibitors etc. (20). A total of 196 eligible patients were categorized into the TTP group and the ITP group according to the final diagnosis. The patient screening process was illustrated in Figure 1.

### 2.2 Data collection

Diagnostic workup included tests for antiplatelet membrane glycoprotein autoantibodies, antinuclear autoantibody group, antiphospholipid antibodies, hepatitis virus serology, thyroid function tests, serum immunoglobulin levels, serum thrombopoietin levels, and genetic testing (20). All diagnoses were centrally assessed by a minimum of one chief physician from the haematology department and one laboratory technician, both of whom were uninformed of other outcomes. Demographic information, fundamental anthropometric data, medical history, clinical characteristics and laboratory test results (mainly blood routine test results), past medical history including that of hypertension, diabetes and other disorders of the patients were collected within 24 h after admission. SII, PLR, PPN, and body mass index (BMI) were calculated according to the following calculation formulas: SII = (neutrophil count × platelet count) / lymphocyte count; PLR = platelet count/lymphocyte count; PP*N* = neutrophil count × platelet count; BMI = weight (kg) / height2 (m2).

### 2.3 Statistical analysis

Continuous variables were presented as mean ± standard deviation (SD) or median (interquartile range, IQR), with normality assessed using the Kolmogorov Smirnov test. Between group comparisons were conducted using t-tests or Mann Whitney U tests, while categorical variables were analyzed with chisquare tests. A two-tailed *p*-value < 0.05 was considered statistically significant. Univariate logistic regression analysis was employed to identify risk factors associated with TTP. All statistical analyses were performed using SPSS software version 21.0.

Feature selection was based on both clinical relevance and statistical significance, with a particular focus on inflammatory markers (SII, PLR, and PPN). These markers have been implicated in thrombosis and immune dysregulation, making them physiologically relevant for distinguishing TTP from ITP. To our knowledge, this was the first study to integrate these inflammatory indices into a machine learning model for TTP diagnosis.

To develop and validate a diagnostic model, data from 196 patients were randomly divided into a training set (90%) and a test set (10%). To mitigate overfitting from limited sample size, we implemented 10-fold cross-validation (stratified by class) with fixed random seed (500). Eight machine learning models were implemented, including Naïve Bayes, XGBoost, Support Vector Machine (SVM), Random Forest, K-Nearest Neighbors (KNN), Gradient Boost Trees, Decision Tree, and Logistic Regression (21, 22). All models were applied to the binary classification task of differentiating TTP from ITP. Model performance was averaged across folds using receiver operating characteristic curve (AUC), recall, F1 score, accuracy, and confusion matrix. All machine learning models were implemented in Python (version 3.11.10) using PyCharm as the development environment. Confusion matrices were generated to compare predicted and actual labels, with grid values representing true negatives (TN), false positives (FP), false negatives (FN), and true positives (TP), thereby enabling direct assessment of each model’s sensitivity and specificity.

Given the urgent need for early TTP diagnosis, AUC was prioritized to minimize false negatives and reduce the risk of missed diagnosis. Model risk factors were visualized using nomograms, model reliability was assessed using calibration curves, and the net clinical benefit of applying the model to decision making was determined using decision curve analysis (DCA) (23).

After comprehensive evaluation, we selected logistic regression as the final model because it achieved the highest balance between accuracy (0.78), interpretability, and clinical feasibility. The feature contribution was visualized using SHAP values. Unlike black-box models such as XGBoost or SVM, logistic regression can directly calculate risk scores, making it more suitable for real world clinical practice.

## 3 Results

### 3.1 Patient characteristics

This study consisted of 196 patients, including 118 females (60.20%) and 78 males (39.80%), with an average age of 47.60 ± 16.74 years, and a BMI of 23.20 ± 3.44. Among the participants, 28 had a history of smoking (14.29%), 14 had a history of alcohol consumption (7.14%), 37 had diabetes (18.88%), 41 had hypertension (20.92%), and 8 had coronary heart disease (4.08%). The analysis of essential laboratory test results (platelet count, lymphocyte count, neutrophil count) indicated that SII ranged from 192.51 ± 221.00, PPN ranged from 224.86 ± 209.99, and PLR ranged from 30.13 ± 32.96. Table 1 enumerates specific demographic and clinical factors.

**Table 1:** Characteristics of the patients.

Variable	Safety analysis set (*n* = 196)
Sex, *n* (%)	
Male	78 (39.80%)
Female	118 (60.20%)
Age (years)	47.60 ± 16.74
Height (cm)	161.48 ± 7.48
Weight (kg)	60.55 ± 10.16
BMI	23.20 ± 3.44
Smoker, n (%)	
No	168 (85.71%)
Yes	28 (14.29%)
Alcohol drinker	
No	182 (92.86%)
Yes	14 (7.14%)
Diabetes	
No	159 (81.12%)
Yes	37 (18.88%)
Hypertension	
No	155 (79.08%)
Yes	41 (20.92%)
Coronary heart disease	
No	188 (95.92%)
Yes	8 (4.08%)
Laboratory test index	
Neutrophil count(×10^9^ /L)	7.02 ± 4.52
Monocyte count^*^ (× 10^9^ /L)	0.56 ± 0.40
Lymphocyte count(×10^9^ /L)	1.52 ± 0.79
Platelet count(×10^9^ /L)	34.78 ± 30.00
SII^*^	192.51 ± 221.00
PPN	224.86 ± 209.99
PLR^*^	30.13 ± 32.96

*Means non-normally distributed data.

The patient’s baseline characteristics were shown in Table 2. Among them, 54 were patients with TTP and 142 were patients with ITP. Parameters such as body weight (*p* = 0.023), BMI (*p* = 0.018), neutrophil count (*p* = 0.043), platelet count (*p* < 0.001), SII (*p* < 0.001), PPN (*p* < 0.001), and PLR (*p* < 0.001) exhibited significant differences between the ITP group and the TTP group (*p* < 0.05). Although the mean BMI was higher in the TTP group, there was substantial overlap in the distributions between groups, suggesting that BMI alone may not reliably distinguish TTP from ITP and should be considered in combination with inflammatory indices.

**Table 2:** Baseline characteristics of two groups.

Variable	ITP (*n* = 142)	TTP (*n* = 54)	*P*
Sex			0.325
Male	53 (37.32%)	25 (46.30%)	
Female	89 (62.68%)	29 (53.70%)	
Age (years)	46.58 ± 16.33	50.30 ± 17.66	0.182
Height (cm)	161.69 ± 7.48	160.93 ± 7.52	0.524
Weight (kg)	61.61 ± 9.84	57.79 ± 10.54	0.023
BMI	23.56 ± 3.40	22.26 ± 3.38	0.018^*^
Smoker			0.579
No	120 (84.51%)	48 (88.89%)	
Yes	22 (15.49%)	6 (11.11%)	
Alcohol drinker			0.761
No	131 (92.25%)	51 (94.44%)	
Yes	11 (7.75%)	3 (5.56%)	
Diabetes			0.777
No	114 (80.28%)	45 (83.33%)	
Yes	28 (19.72%)	9 (16.67%)	
Hypertension			0.386
No	115 (80.99%)	40 (74.07%)	
Yes	27 (19.01%)	14 (25.93%)	
Coronary heart disease			1
No	136 (95.77%)	52 (96.30%)	
Yes	6 (4.23%)	2 (3.70%)	
Laboratory test index			
Neutrophil count	6.66 ± 4.74	7.98 ± 3.76	0.043
Monocyte count	0.53 ± 0.37	0.65 ± 0.47	0.112
Lymphocyte count	1.57 ± 0.78	1.36 ± 0.80	0.101
Platelet count	28.35 ± 22.15	51.67 ± 40.05	< 0.001^*^
SII	129.17 ± 119.18	359.09 ± 320.76	< 0.001^*^
PPN	169.96 ± 168.27	369.23 ± 240.00	< 0.001^*^
PLR	21.99 ± 18.57	51.53 ± 49.34	< 0.001^*^

P* < 0.05 means statistical significance.

Given that BMI calculation incorporates weight and height, and combined inflammatory index calculation encompasses neutrophil, platelet, and lymphocyte count parameters, in order to avoid duplication, the subsequent studies excluded weight, height, and individual laboratory indicators to prevent redundancy.

### 3.2 Risk factors

Table 3 illustrates the compositional differences of variables between the two groups. Significant differences in BMI, SII, PPN, and PLR (*p* < 0.05) between the groups were determined as risk factors. Among them, BMI was a protective factor (OR = 0.89, 95% CI (0.80, 0.98), *p* = 0.019), whereas SII, PPN, and PLR were identified as risk factors [OR = 1.01, 95% CI (1.00, 1.01), OR = 1.00, 95% CI (1.00, 1.01), OR = 1.03, 95% CI (1.02, 1.05), respectively].

**Table 3:** The univariate logistic regression analysis.

Variables	OR (95%CI)	*P*
Sex		
Male	Reference	
Female	0.69 (0.37, 1.31)	0.258
Age (years)	1.01 (0.99, 1.03)	0.165
Height (cm)	0.99 (0.95, 1.03)	0.52
Weight (kg)	0.96 (0.93, 0.99)	0.02
BMI smoker	0.89 (0.80, 0.98)	0.019^*^
No	Reference	
Yes	0.69 (0.24, 1.74)	0.452
Alcohol drinker		
No	Reference	
Yes	0.73 (0.15, 2.48)	0.632
Diabetes		
No	Reference	
Yes	0.82 (0.34, 1.83)	0.643
Hypertension		
No	Reference	
Yes	1.49 (0.70, 3.11)	0.297
Coronary heart disease		
No	Reference	
Yes	0.91 (0.12, 4.27)	0.916
Laboratory test index		
SII	1.01 (1.00, 1.01)	< 0.001^*^
PPN	1.00 (1.00, 1.01)	< 0.001^*^
PLR	1.03 (1.02, 1.05)	< 0.001^*^

^*^P < 0.05 means statistical significance.

### 3.3 Machine learning models and performance evaluation

**Figure 2:**
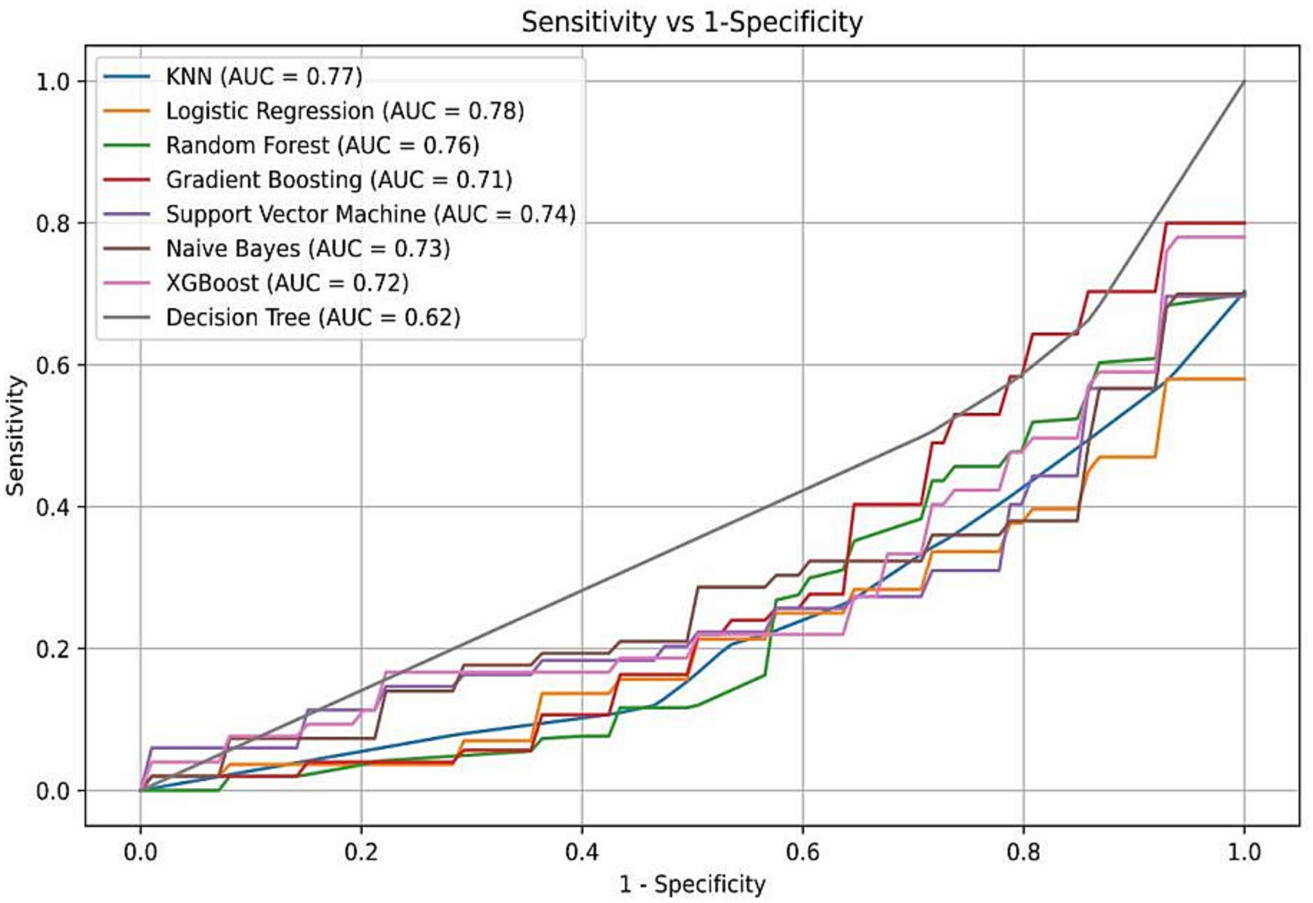
Receiver operating characteristic (ROC) curves of eight machine learning models. Logistic regression demonstrated the highest AUC (0.78), followed by Random Forest, XGBoost, and Naive Bayes. The yellow curve represents the logistic regression model, which was ultimately selected for its performance and interpretability.

**Figure 3:**
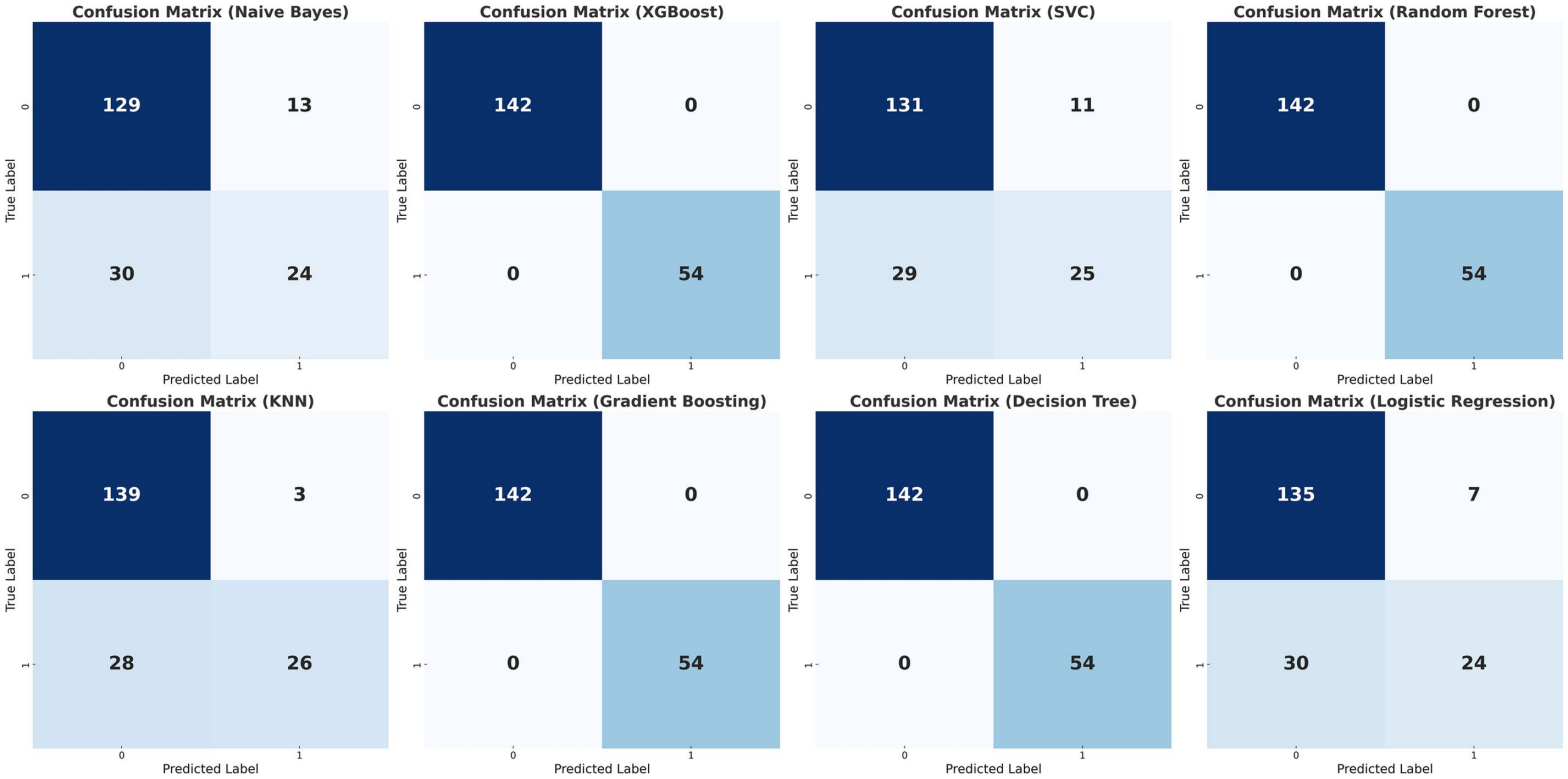
Confusion matrices of the eight classification models. Confusion matrices of the eight machine learning models (Naïve Bayes, XGBoost, Support Vector Classifier, Random Forest, K-Nearest Neighbors, Gradient Boosting, Decision Tree, and Logistic Regression) for differentiating TTP from ITP. Columns represent predicted labels (0 = ITP, 1 = TTP) and rows represent true labels, with grid values showing case counts of true negatives (TN, upper left), false positives (FP, upper right), false negatives (FN, lower lef), and true positives (TP, lower right). The matrices were generated by 10-fold cross-validation, and logistic regression demonstrated the most balanced performance across true positives and true negatives.

The data were randomly allocated to the training set and the test set in a 9:1 ratio utilizing Python. The internal validation employed 10-fold cross-validation with a seed number of 500 to guarantee the consistency of all parameters. Eight machine learning models were constructed (Table 4): Naive Bayes, Extreme Gradient Boosting (XGBoost), Support Vector Machine (SVM), Random Forest, K-Nearest Neighbors (KNN), Gradient Boosting Tree, Decision Tree, and Logistic Regression. All models were applied to the binary classification task of differentiating TTP from ITP. Together with Area under curve (AUC), recall, F1 score, accuracy, and confusion matrix were established as performance evaluations. The receiver operator characteristics (ROC) curve and confusion matrix was illustrated (Figures 2, 3) to enhance the intuitiveness and clarity of the model’s prediction ability.

**Table 4:** Machine learning development.

Methods	AUC	Recall	F1	Presicion
Naive Bayes	0.7281	0.4667	0.5184	0.6731
XGBoost	0.7192	0.4667	0.4877	0.5644
SVM	0.7372	0.3667	0.4442	0.6283
Random Forest	0.7573	0.3733	0.4252	0.5429
KNN	0.7702	0.3833	0.4636	0.6833
Gradient Boost Trees	0.7141	0.2767	0.3187	0.3867
Decision Tree	0.6195	**0.4867**	0.4554	0.4357
Logistic Regression	**0.7845**	0.4633	**0.5502**	**0.7833**

The columns of the chart are eight machine models: Naive Bayes, XGBoost, SVM, Random Forest, KNN, Gradient Boost Trees, Decision Tree, and Logistic Regression, respectively, and the rows are the performance indicators of these eight machine models, namely AUC, Recall, F1, and Precision. Bold values indicate the highest values across all machine learning models for each metric, highlighting the superior performance of logistic regression.

The diagnostic performance of the eight machine learning models was summarized in the confusion matrices shown in Figure 3. Each matrix illustrates the numbers of true negatives (TN), false positives (FP), false negatives (FN), and true positives (TP), providing a visual complement to summary metrics. While several models, such as Random Forest, XGBoost, and Decision Tree, demonstrated relatively high true negative counts, they yielded few true positives, resulting in higher false negatives and thus lower recall and F1 scores—an especially critical limitation in clinical contexts where minimizing missed TTP diagnoses is essential. Logistic regression, by contrast, achieved the most balanced distribution between true positives and true negatives, consistent with its superior performance across multiple evaluation metrics. Specifically, it yielded the highest AUC (0.78; 95% CI: 0.71–0.85), recall (0.46), F1 score (0.55), and accuracy (0.78), outperforming more complex classifiers including SVM, Gradient Boosting, and KNN (Figure 2; Table 4). These results, supported by both numerical metrics and the confusion matrix visualization, highlight logistic regression as the most clinically reliable and interpretable model for early TTP risk prediction. Notably, the discrimination between TTP and ITP was primarily driven by composite inflammatory markers (SII, PPN, and PLR), with logistic regression leveraging these features to achieve the most reliable performance.

**Figure 4:**
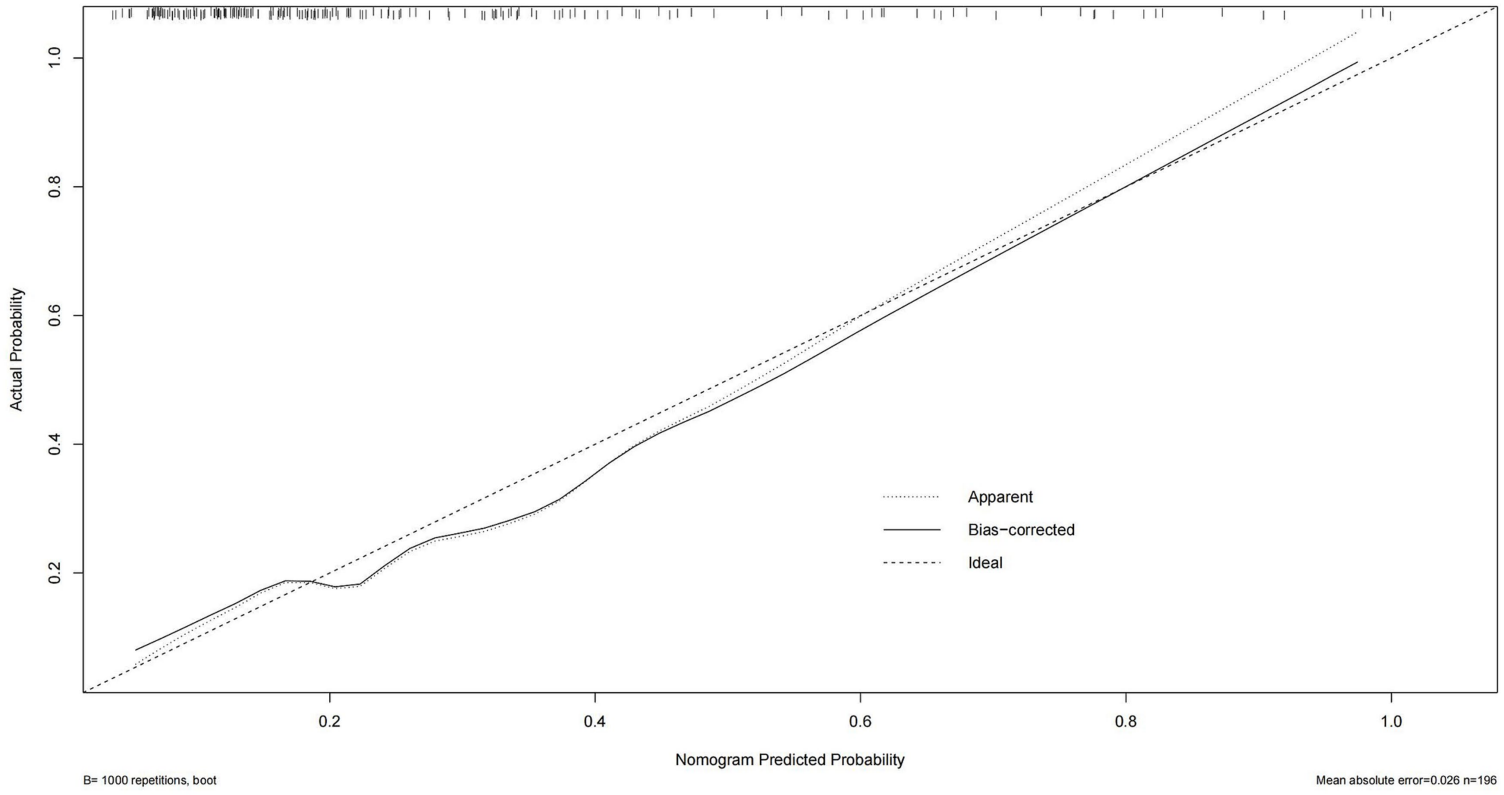
Calibration curve of the logistic regression model. The predicted probability of TTP is plotted against the observed proportion. A curve closer to the diagonal reference line indicates better calibration and model reliability in predicting actual outcomes.

**Figure 5:**
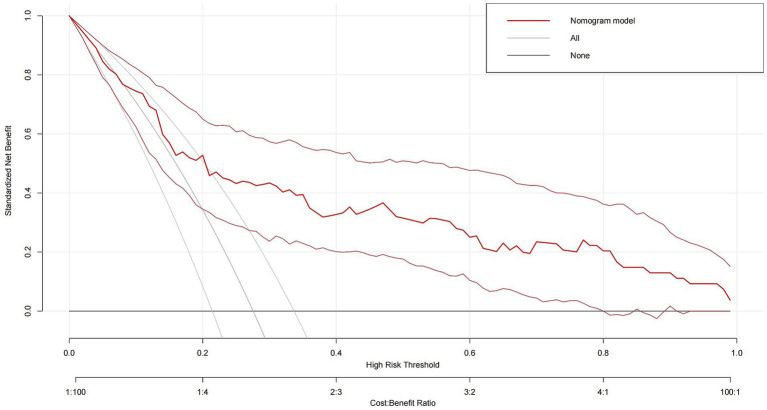
Decision curve analysis (DCA) of the logistic regression model. The x-axis represents threshold probabilities, and the y-axis shows the net clinical benefit. The model demonstrates a consistent net benefit across a range of threshold values, suggesting its potential utility in guiding clinical decision-making for TTP risk stratification.

**Figure 6:**
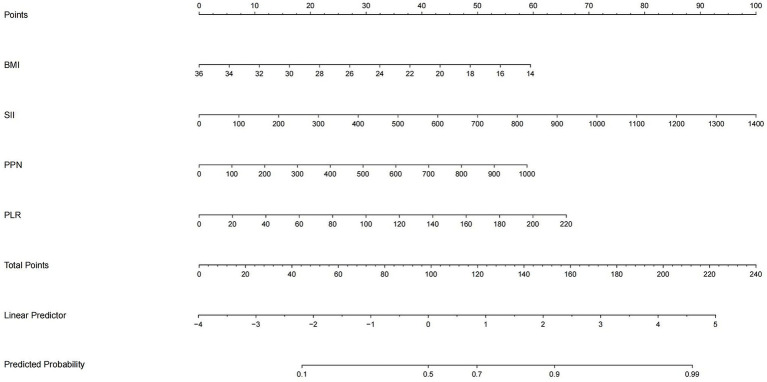
Nomogram based on logistic regression output. The nomogram incorporates four key predictors: SII, PPN, PLR, and BMI. For each patient, the score of each variable is read on its respective axis and summed to yield a total score, which corresponds to the predicted probability of TTP. This tool allows intuitive and individualized clinical risk estimation.

Consequently, with the logistic regression model, the fitting curve was generated by 1,000 repeated extractions. A calibration curve (Figure 4) and a Decision Curve Analysis (DCA) curve (Figure 5) were constructed. The results indicated that the calibration curve’s real fit was consistent with the ideal fit, demonstrating that the predicted probability matched the actual probability. The clinical decision curve exhibited a substantial clinical benefit rate, further elucidating the applicability of this approach in clinical settings. So, a nomogram (Figure 6) prediction model was developed to visualize risk factors and assess TTP risk. The four variables were assigned the four scores in the nomogram. The probability could be directly obtained by adding the scores of each predictor, further visualizing the clinical data of high risk TTP patients, boosting diagnostic accuracy.

### 3.4 Visualization of feature significance

**Figure 7:**
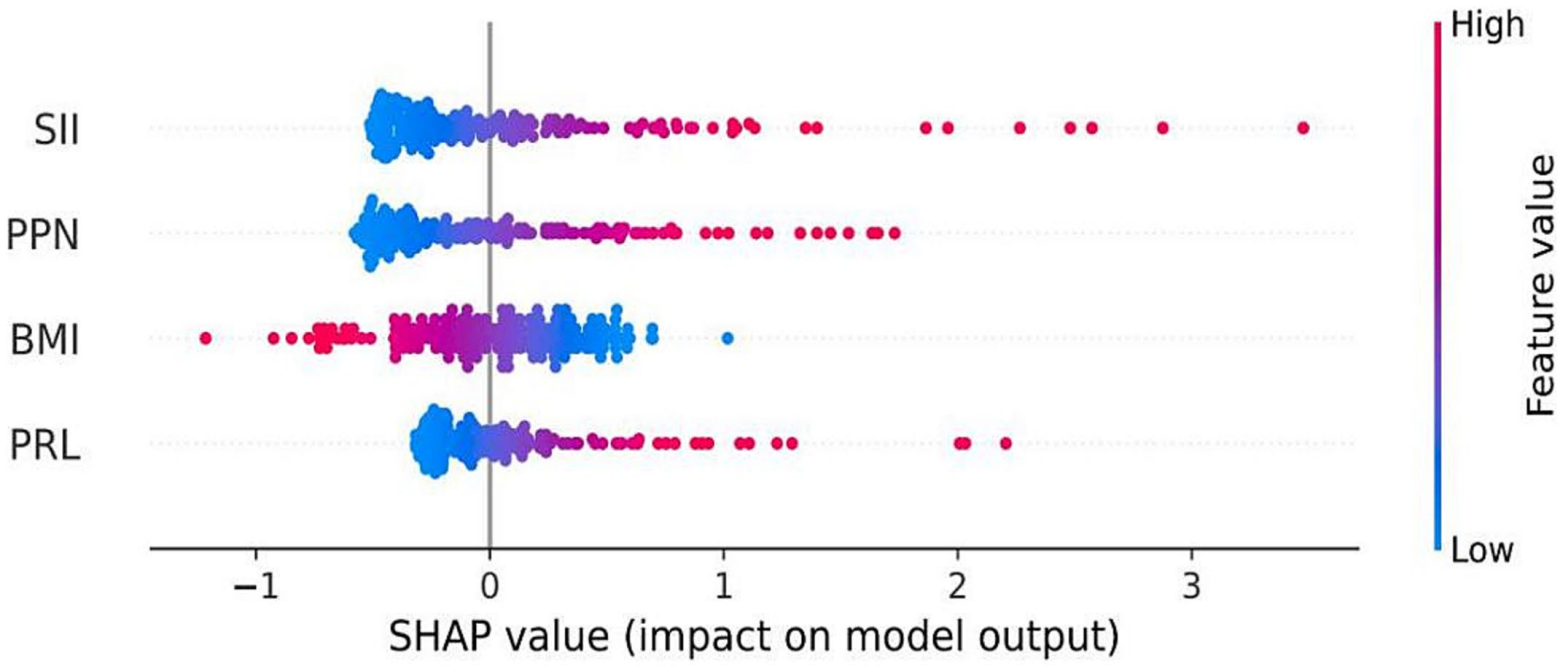
SHAP summary plot for model interpretation. Shapley Additive Explanations (SHAP) visualize the contribution of each feature to the model output. Red dots represent high values of a variable, and blue dots represent low values. Variables on top (especially SII) had the greatest impact on model predictions.

**Figure 8:**
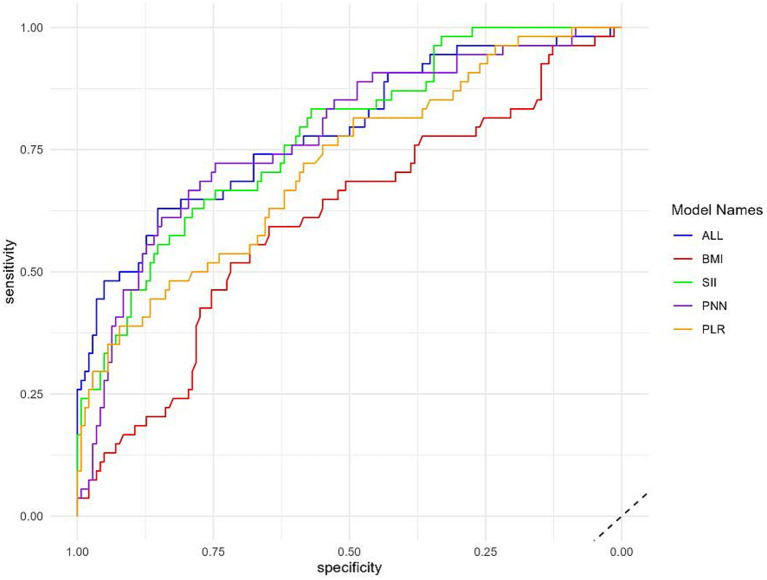
Variable importance plot. The figure displays the relative importance of the top four features contributing to the model. SII ranked highest, followed by PPN, PLR, and BMI, confirming the value of composite inflammatory indices in distinguishing TTP from ITP.

The Shapley additive explanation (SHAP) and AUC were used to visually display the influence of the selected variables on the diagnosis of TTP. Figure 7 illustrates the classification of the four variable features in the model, plotting points of different colors corresponding to each feature variable. It showed that SII is the main risk factor affecting the diagnosis of TTP, and confirms that SII is the most influential feature for distinguishing TTP from ITP, and its predictive ability exceeds that of PPN and PLR. In addition, ROC calculation (Figure 8) showed that the ROC of SII = 0.777 (95% CI = 0.706, 0.849), which exceeds the ROC of PPN 0.776 (95% CI = 0.701, 0.851). SII was identified as the most influential predictor in our model, consistent with its role in systemic inflammation and platelet activation.

## 4 Discussion

Thrombotic thrombocytopenic purpura (TTP) is a life threatening thrombotic microangiopathy that requires rapid diagnosis and timely intervention. While therapeutic plasma exchange (TPE) has significantly reduced mortality to 10–20% (24–26), the current reliance on ADAMTS13 activity testing presents challenges, particularly in resource limited settings where accessibility and turnaround time are significant barriers (27). Given these limitations in current diagnostic methods, our study provides a potential alternative for rapid screening. Our study addresses this gap by developing a machine learning based predictive model that integrates routine inflammatory markers (SII, PPN, and PLR) to achieve rapid TTP risk stratification, demonstrating a robust diagnostic performance (AUC = 0.78).

Current diagnostic pathways for TTP rely heavily on ADAMTS13 assays, which may require 24–72 h for results (28–30). By contrast, our model enabled real time risk estimation using routine blood tests, facilitating early identification of high risk patients and prompting expedited confirmatory testing or preemptive intervention. This is particularly relevant in settings where ADAMTS13 assays are unavailable or delayed. By providing an accessible and cost effective screening tool, this model had the potential to streamline clinical workflows and improve patient outcomes, particularly in resource limited environments.

Although the PLASMIC score demonstrated high diagnostic accuracy (AUC = 0.92) and is widely used in clinical practice, it is primarily derived from routinely available clinical and laboratory variables—such as platelet count, hemolysis markers, renal function, and underlying malignancy—and does not depend on ADAMTS13 activity testing (31, 32). In this regard, our model is not intended to replace the PLASMIC score but rather to serve as a complementary tool. Unlike the PLASMIC score, which integrates a broader set of variables, our approach was purposefully restricted to three simple inflammatory composites (SII, PPN, and PLR) together with BMI. While other routine laboratory measures (e.g., INR, bilirubin, reticulocyte count, haptoglobin) could also provide diagnostic value, our focus on inflammatory indices offers a physiologically grounded, low-cost, and universally accessible framework. This design makes the model particularly suitable for rapid triage in emergency or resource-limited settings where comprehensive laboratory panels or ADAMTS13 assays may be unavailable or delayed.

Previous ML-based diagnostic studies in hematology and related fields often rely on high-dimensional inputs (e.g., electronic health record variables, imaging features, or specialized biomarkers) and complex classifiers, which may achieve high apparent accuracy but face challenges with calibration, generalizability, and clinical adoption—particularly in resource-limited settings (21, 22, 33–39). In contrast, our model was intentionally designed to use only routine, low-cost inflammatory composites (SII, PPN, and PLR) together with BMI, thereby anchoring prediction in well-established pathophysiological processes while ensuring scalability and accessibility where ADAMTS13 testing is delayed or unavailable (5, 9–13, 24–27, 31, 32). This design highlights the novelty of our study and emphasizes its suitability for rapid, point-of-care screening.

Although tree-based models such as XGBoost and Random Forest achieved comparable AUC values in our dataset, their “black-box” nature raises concerns about interpretability and clinical uptake. By contrast, logistic regression provides direct risk quantification through odds ratios and can be readily implemented as a nomogram-based prediction tool (Figure 6). This transparency supports real-world clinical decision making and aligns with prior recommendations advocating interpretable models for clinical predictive applications (40).

Notably, SII emerged as the most significant predictor of TTP risk, consistent with its established role in systemic inflammation and platelet activation (31, 32). Elevated SII has been extensively studied as a prognostic marker in various conditions, including malignancies, cardiovascular diseases, and autoimmune disorders, where it serves as an indicator of heightened inflammatory activity and immune dysregulation. In the context of TTP, the role of inflammation in disease pathophysiology is well established (9–13), with evidence suggesting that neutrophil activation and platelet aggregation contribute to microvascular thrombosis (5). Increased SII may indicate excessive neutrophil driven inflammation and platelet activation, both of which are key contributors to TTP related thrombotic events. Furthermore, previous studies have linked elevated SII levels with increased risk of endothelial dysfunction, hypercoagulability, and microvascular occlusion hallmarks of TTP progression. Given its simplicity and strong pathophysiological relevance, SII represents a promising biomarker for identifying high risk TTP patients and may complement existing diagnostic strategies by providing a rapid and cost effective risk assessment tool. Along with PPN and PLR, these composite inflammatory markers served as the core features for distinguishing TTP from ITP in our models, with logistic regression leveraging them to achieve the most balanced and clinically practical diagnostic performance.

This study has several limitations that warrant consideration. First, it was a single center, retrospective analysis with a relatively small sample size, especially for TTP cases. Although TTP is a rare disorder, the limited number of events may restrict the generalizability and statistical power of the findings. Future multi-center, large-scale studies are necessary to validate the model across more diverse populations and clinical settings. Second, the model was only internally validated using cross validation within the existing dataset. While 10-fold cross-validation helps mitigate overfitting, external validation on independent cohorts is essential to assess the model’s real world applicability and robustness. Third, the model exclusively included inflammatory markers derived from routine blood tests (SII, PPN, PLR) and BMI, without integrating other potential clinical variables such as LDH, bilirubin, or creatinine levels. Although this was done intentionally to maintain simplicity and accessibility, it may limit the model’s predictive accuracy compared to comprehensive scoring systems like PLASMIC. Fourth, our model does not distinguish between acquired and congenital forms of TTP, which may have distinct inflammatory profiles. Stratified analysis in future studies may provide a more nuanced understanding of the model’s diagnostic performance across TTP subtypes. Lastly, while the model showed promise for early triage, it cannot substitute for definitive ADAMTS13 testing, which remains the gold standard for TTP diagnosis. Our approach is intended as a complementary, rapid screening tool—particularly useful in settings where timely ADAMTS13 results are unavailable. Future studies may also explore incorporating additional routine laboratory parameters to further enhance performance.

## 5 Conclusion

This study developed a machine learning based model incorporating inflammatory indices for TTP risk prediction. Logistic regression demonstrated optimal performance, with SII emerging as the most influential predictor. This model had the potential to improve early TTP diagnosis, reduce diagnostic delays, and facilitate timely intervention, particularly in resource limited settings. Further validation through prospective, multi-center studies is warranted to confirm clinical applicability and integration into routine practice. With further validation, this approach may be integrated into routine clinical workflows to facilitate early and accessible TTP diagnosis.

## Data Availability

The datasets generated and analyzed during the current study are not publicly available due to institutional restrictions and patient privacy considerations. However, de-identified data supporting the conclusions of this article may be made available by the corresponding author upon reasonable request and with appropriate ethical approvals. The raw data supporting the conclusions of this article will be made available by the authors, without undue reservation.
