# Supplementary material for: Systemic immune inflammation index guides machine learning for rapid TTP diagnosis: a retrospective cohort study
Source: Front Med (Lausanne). 2025 Oct 16;12:1599999. doi: 10.3389/fmed.2025.1599999 (PMC12571784; doi:10.3389/fmed.2025.1599999)
Supplement: Supplementary file 1 [file Table_1.docx]

# Supplementary Table 1. Simulated Sample of TTP and ITP Patient Data Used for Model Construction

| Patient_ID | Diagnosis | SII | PLR | PPN | BMI |
| --- | --- | --- | --- | --- | --- |
| Sim_1 | TTP | 666 | 50.2 | 80.3 | 27.4 |
| Sim_2 | ITP | 1438 | 248.4 | 61.8 | 23.6 |
| Sim_3 | TTP | 530 | 173.5 | 130.4 | 21.9 |
| Sim_4 | TTP | 287 | 172.3 | 99.7 | 18.2 |
| Sim_5 | TTP | 1323 | 51.4 | 177.8 | 20.3 |
| Sim_6 | ITP | 1071 | 54.6 | 110.7 | 20.4 |
| Sim_7 | TTP | 330 | 155.0 | 161.8 | 24.8 |
| Sim_8 | TTP | 969 | 130.0 | 138.4 | 24.1 |
| Sim_9 | TTP | 543 | 59.3 | 108.6 | 26.3 |
| Sim_10 | ITP | 1005 | 244.8 | 51.7 | 19.7 |
